# Thyroid Paraganglioma: A Rare Manifestation of Paraganglioma Syndrome Associated With Pathogenic Variant in *SDHD*

**DOI:** 10.1210/jcemcr/luae135

**Published:** 2024-08-16

**Authors:** Valentina D Tarasova, Kelara Samuel, Caitlin McMullen, Sergiy Kushchayev, Juan C Hernandez Prera, Colleen Veloski

**Affiliations:** Department of Head and Neck-Endocrine Oncology, Moffitt Cancer Center, Tampa, FL 33612, USA; Section of Internal Medicine, University of South Florida, Tampa, FL 33612, USA; Department of Head and Neck-Endocrine Oncology, Moffitt Cancer Center, Tampa, FL 33612, USA; Department of Radiology, Moffitt Cancer Center, Tampa, FL 33612, USA; Department of Pathology, Moffitt Cancer Center, Tampa, FL 33612, USA; Department of Head and Neck-Endocrine Oncology, Moffitt Cancer Center, Tampa, FL 33612, USA

**Keywords:** thyroid paraganglioma, *SDHD*

## Abstract

Evaluation of an incidentally discovered indeterminate thyroid nodule (TN) in a previously healthy 59-year female led to diagnosis of thyroid paraganglioma (TPGL) and subsequently hereditary succinate dehydrogenase complex subunit D (*SDHD)*-related multifocal head and neck paragangliomas (PGLs). An ultrasound-guided fine needle aspiration (FNA) biopsy of the 1.7-cm TN was nondiagnostic and core biopsy was suspicious for papillary thyroid carcinoma. Pathology slides reviewed at tertiary center showed neuroendocrine neoplasm consistent with PGL. Her 24-hour urinary catecholamines and metanephrines were normal. Given the diagnosis of TPGL, genetic testing was recommended, which identified a pathogenic variant in *SDHD* (c.242C > T(p.P81L). Gallium-68-DOTATATE PET/CT revealed multifocal areas of increased somatostatin receptor expression from the skull base to thoracic inlet. Magnetic resonance imaging of the brain/neck showed multiple PGLs (right jugular, carotid, thyroid, left vagal, left level II, and superior mediastinal), all measured up to 1.7 cm. The right jugular PGL was treated with external beam radiation therapy of 3000 cGy. All PGLs remained stable and asymptomatic at 22-month follow-up imaging. TPGL should be considered in the differential diagnosis of a hypervascular TN in patients with *SDH*x-related pheochromocytoma-PGL syndromes and when such lesions with indeterminate cytology are encountered in patients with no known history of *SDHx*-mutation or syndrome.

## Introduction

Thyroid paragangliomas (TPGL) are exceptionally rare, representing 0.5% of all paragangliomas (PGL) with about 80 cases reported to date [1, 2]. Preoperative diagnosis of TPGL is challenging requiring high clinical suspicion to drive additional evaluation in selected patients [1, 2]. Here we report a case of a TPGL presenting as an indeterminate thyroid nodule (TN) ultimately leading to the diagnosis of multifocal head and neck PGL syndrome due to a pathogenic variant in succinate dehydrogenase complex subunit D (*SDHD*).

## Case Presentation

A 59-year-old generally healthy female individual was initially seen at a tertiary referral center for consideration of thyroidectomy for an indeterminate TN discovered incidentally on neck ultrasound ordered for evaluation of a palpable neck mass. TN was asymptomatic and palpable on physical exam.

## Diagnostic Assessment

**Figure 1.**
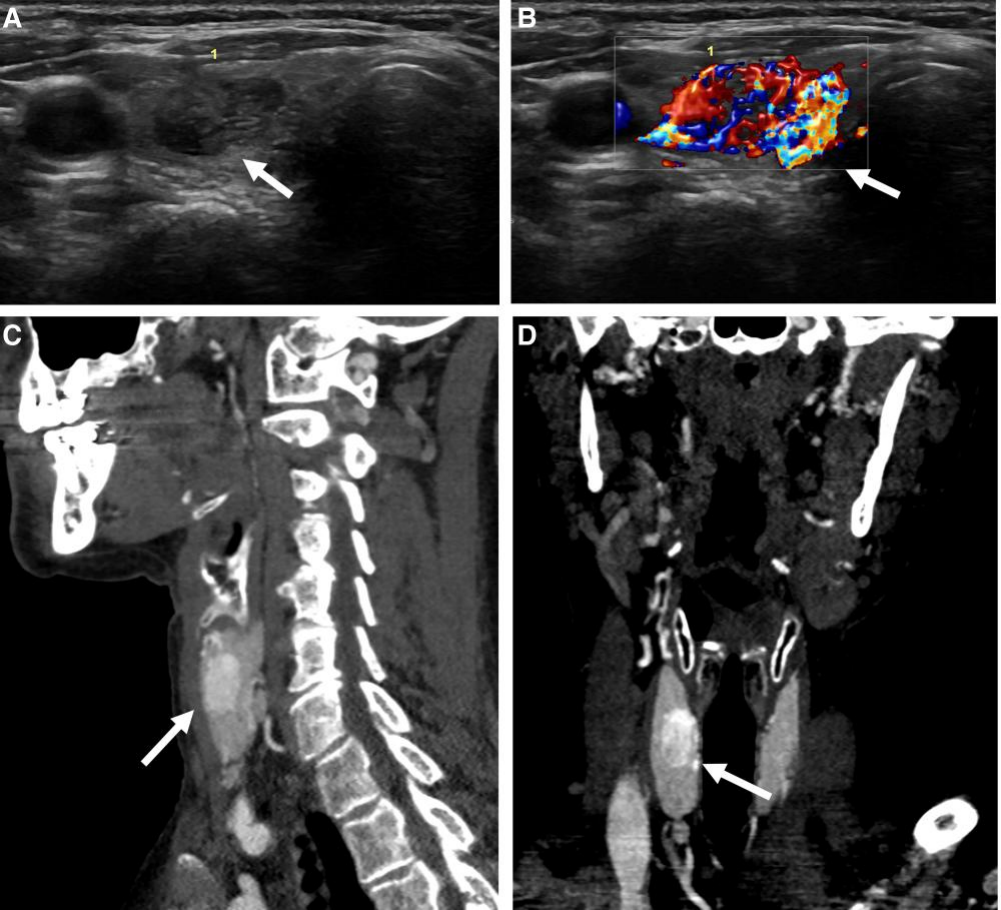
Thyroid paraganglioma images. A and B: Thyroid ultrasound revealed a 1.7 × 0.9 × 1.2 cm right solid, hypoechoic, hypervascular, wider than tall thyroid nodule with lobulated margins and without evidence of extrathyroidal extension. C and D thyroid paraganglioma in the right thyroid lobe on computed tomography (CT) neck with contrast.

Neck and thyroid ultrasound showed multiple nonspecific bilateral lymph nodes/neck masses measured up to 1.8 × 0.7 × 0.7 cm, hypoechoic without fatty hila, and a 1.7 × 0.9 × 1.2 cm right interpolar markedly hypoechoic, hypervascular solid TN (TIRAD 5) (Fig. 1). The patient did not have symptoms related to the TN or lymph nodes/masses in the neck; no sensation of a mass in the neck or dysphagia. Thyroid function tests were within normal range: thyroid-stimulating hormone (TSH) 1.6 mIU/L (1.6 mcIU/mL) (normal reference range 0.5-4.5 mIU/L; 0.5-4.5 mcIU/mL), free thyroxine (FT4) 0.78 ng/dL (10 pmol/L) (normal reference range 0.7-1.48 ng/dL; 9-19 pmol/L).

**Figure 2.**
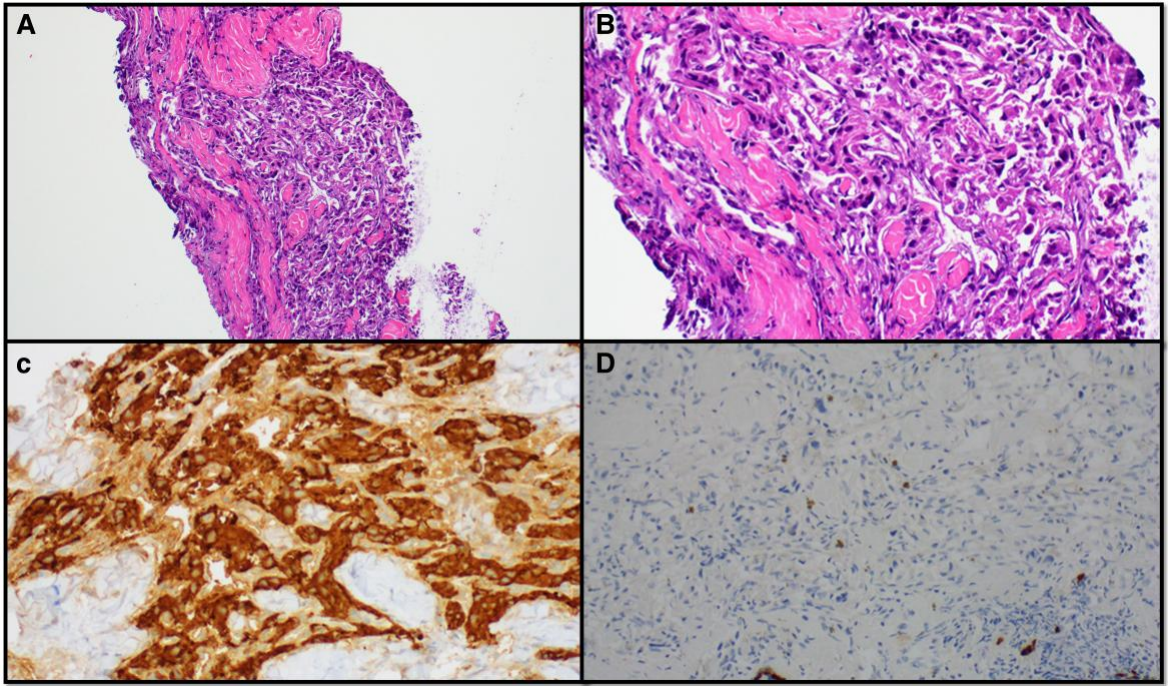
Pathology. A: Right thyroid nodule core biopsy showing cellular proliferation of epithelioid to spindle shaped cell arranged in a nested pattern. B: Tumor cells with eosinophilic cytoplasm with indistinct cell borders and oval monotonous nuclei lacking nuclear atypia or necrosis. C: Tumor cells diffusely positive for synaptophysin. D: Tumor cells are negative for cytokeratin.

An ultrasound-guided FNA biopsy of a 1.7 cm TN was nondiagnostic, Bethesda I. A subsequent core biopsy was initially interpreted as suspicious for papillary thyroid carcinoma. Pathology slides reviewed at the tertiary center confirmed a nondiagnostic sample (Bethesda I) on cytopathology, but the core biopsy diagnosis was revised. The core biopsy showed cellular proliferation of epithelioid to spindle shaped cells arranged in a nested pattern (Fig. 2). The tumor cells had eosinophilic cytoplasm with indistinct cell borders and oval monotonous nuclei lacking nuclear atypia or necrosis. At the periphery of the proliferation, there was a rim of thyroid follicular cells. Immunohistochemical studies revealed that the tumor cells were strongly positive for synaptophysin, chromogranin, and GATA3, while S100 stain highlighted sustentacular cells. The tumor cells were negative for cytokeratin, TTF1, thyroglobulin, calcitonin, monoclonal CEA. The overall findings were diagnostic of a PGL.

Serum calcitonin and 24-hour urinary catecholamines and metanephrines were within normal limits (calcitonin 4.4 pg/mL [1.3 pmol/L] [normal reference range 0.0-5.1 pg/mL; 0-1.5 pmol/L]). The 24-hour urinary catecholamine and metanephrine values were: norepinephrine 32 ug/d (174 nmol/d) (normal reference range 14-120 ug/d; 76-665 nmol/d), epinephrine 8 ug/d (43 nmol/d) (normal reference range 1-14 ug/d; 5-76 nmol/d), dopamine 262 ug/d (1430 nmol/d) (normal reference range 71-485 ug/d; 387-1710 nmol/d), metanephrine 135 ug/d (737 nmol/d) (normal reference range 36-229 ug/d; 196-1250 nmol/d), normetanephrine 218 ug/d (1190 nmol/d) (normal reference range 95-650 ug/d; 518-3548 nmol/d). There was no family history of pheochromocytoma or PGL. Germline genetic testing using peripheral blood lymphocytes detected a pathogenic variant in *SDHD* (c.242C > T (p.P81L)). First-degree relatives were recommended to be tested (mother was deceased, 88-year-old father elected not to be tested). The patient's daughter was negative and her generally healthy son elected not to be tested. The patient's sister was found to have a pathogenic variant in *SDHD* (c.242C > T (p.P81L) without clinical manifestation of the condition; all 3 of her children tested negative. The patient's generally healthy brother declined testing.

**Figure 3.**
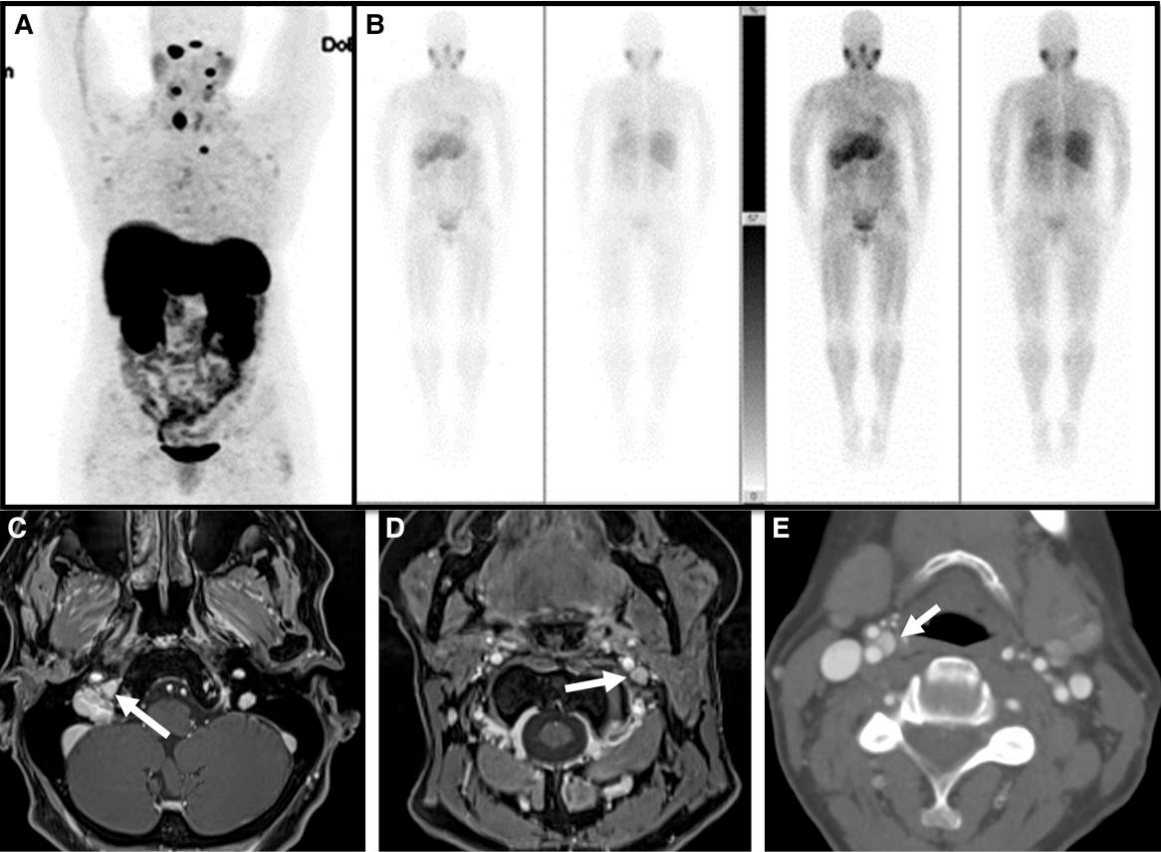
Images of multiple head and neck paragangliomas. A: Gallium-68 (68Ga)-DOTATATE positron emission tomography/computed tomography (PET/CT) revealed multifocal areas of increased somatostatin receptor expression from the skull base to the left thoracic inlet (right and left skull base, left level IIA, right and left level III, right thyroid lobe, and left prevascular superior mediastinum). B: Metaiodobenzylguanidine (MIBG) nuclear medicine scan revealed no radiotracer accumulation in the lesions. C, D, E: Magnetic resonance imaging (MRI) of the neck with and without contrast showed right jugular skull base 1.7 × 1.2 cm paraganglioma (C), 0.6 × 0.5 cm left vagal paraganglioma (D), and 0.6 × 0.6 cm right carotid paraganglioma (E).

Gallium-68 (68Ga)-DOTATATE positron emission tomography/computed tomography (PET/CT) revealed multifocal areas of increased somatostatin receptor expression from the skull base to the left thoracic inlet, which included lesions at right and left skull base, left level IIA, right and left level III, right thyroid lobe, and left prevascular superior mediastinum (Fig. 3A). I-123 metaiodobenzylguanidine (MIBG) nuclear medicine scan revealed no radiotracer accumulation in the lesions (Fig. 3B).

Magnetic resonance imaging (MRI) of the brain and neck with and without contrast in concordance with computed tomography (CT) of the neck with contrast showed multiple PGLs (1.7 × 1.2 cm right jugular skull base, 0.6 × 0.6 cm right carotid, 0.6 × 0.5 cm left vagal, and 1.3 × 0.9 cm right thyroid), 1.1 × 0.6 cm left level II, and 0.4 × 0.4 cm left superior mediastinal (Fig. 3C-3E).

## Treatment

Surgery, systemic therapy, external beam radiation therapy (EBRT), and active surveillance were discussed at the multidisciplinary tumor board. The recommendation was to monitor PGLs including TPGL as they were clinically asymptomatic and multifocal, and to proceed with EBRT to the critically located skull base PGL. The right 1.7-cm skull base PGL was treated with 3000 cGy in 5 fractions over 1 week. The patient tolerated EBRT therapy well with no side effects.

## Outcome and Follow-Up

At 22 months of follow-up, there was no evidence of progression in any of the lesions. There were no new lesions on functional or anatomical imaging (68Ga-DOTATATE PET/CT and CT neck with and without contrast). The patient remained clinically asymptomatic, clinically, and biochemically euthyroid.

## Discussion

Here we present a patient with *SDHD*-related multifocal head and neck PGLs including TPGL discovered during the evaluation of an indeterminate TN. Preoperative diagnosis of TPGL is challenging, therefore most are diagnosed after thyroidectomy [1, 3]. FNA biopsy of TPGL may be nondiagnostic or indeterminate [2]. Therefore, clinical suspicion for TPGL should remain high in patients with pathogenic variants in *SDHx* and highly vascular, markedly hypoechoic TNs on ultrasonography. When clinical suspicion TPGL is high, core biopsy with immunohistochemistry should be considered [3].

True prevalence of *SDH*x-related TPGL is unknown as the germline testing was not routinely obtained on all patients with TPGL. To our knowledge this is the third case report of TPGL that is associated with a pathogenic variant in *SDHD* [4, 5]. Previously reported TPGLs were also associated with pathogenic variants in *SDHB* and *SDHA* (Table 1) [1, 4-6]. Mutation in *SDHD* gene is predominant in head and neck PGLs and has a better prognosis than *SDHB*-related pheochromocytomas/PGLs [7-9].

**Table 1.** SDHx-related thyroid paragangliomas

Case	Age (years)	Sex	Germline pathogenic variant	Nucleotide variation and predicted effect on protein	Reference
1	36	Female	*SDHA*	c.394T > C (p.Trp132Arg)	von Dobschuetz (1)
2	37	Female	*SDHA*	c.1799G > A (p.Arg600Gln)	von Dobschuetz (1)
3	27	Male	*SDHB*	c.530G > A (p.Arg177His)	von Dobschuetz (1)
4	32	Male	*SDHB*	c.201-1339_239delinsAluYb8 p.?	von Dobschuetz (1)
5	32	Female	*SDHB*	392delC	Zantour (6)
6	42	Male	*SDHD*	p.P181L	Sangtian (4)
7	49	Female	*SDHD*	unknown	Raymond (5)
8	59	Female	*SDHD*	c.242C > T (p.Pro81Leu)	Tarasova (this case report)

Abbreviations: *SDHA*, succinate dehydrogenase complex subunit A; *SDHB*, succinate dehydrogenase complex subunit B; *SDHD*, succinate dehydrogenase complex subunit D.

Most head and neck PGLs have an indolent clinical course [10]. Distinguishing between metastatic and multifocal disease in patients with *SDH*x-related pheochromocytoma/PGL syndromes can be difficult since *SDH*x-related PGLs are frequently multifocal. The diagnosis of metastatic PGL is predicated by the identification of the tumor cells in an anatomic location where paraganglia are not normally present (ie, lymph node parenchyma, bone). Given the location of the head and neck PGLs in our patient along path the cervical parasympathetic nerve, the findings are consistent with multifocal cervical disease.

TPGLs arise from inferior laryngeal paraganglia and most commonly present as an asymptomatic TN [3]. Most of the head and neck PGLs, including TPGLs, are nonfunctional; however, biochemical evaluation is indicated [8]. In concordance with previous data, our 24-hour catecholamines and metanephrines were within normal limits [11].

Management of head and neck PGLs depends on the size of the tumor, extent of the disease, symptoms, and rate of progression. Historically, surgery was the gold standard for treatment of head and neck PGLs [12]. Better understanding of the biological behavior of the head and neck PGLs and the possible complications of surgery led to paradigm shift to nonsurgical treatment options: observation and radiation therapy [12, 13]. Most of the reported TPGLs were treated with total or partial thyroidectomy [1]. Data on active surveillance of TPGLs is limited. Our case describes 22-month stability of TPGLs and other head and neck PGLs. Surgery may be considered for solitary, symptomatic, or progressive TPGLs. An observational approach can be appropriate for asymptomatic, nonfunctional, stable TPGLs, that are part of multifocal head and neck PGLs.

## Learning Points

- TPGL should be considered in the differential diagnosis of suspicious hypervascular indeterminate TNs, particularly in patients with *SDH*x-related pheochromocytoma-PGL syndromes.
- Preoperative diagnosis of TPGL is challenging, usually requiring core biopsy with specific stains.
- Active surveillance is an appropriate strategy for small, stable, asymptomatic, nonfunctional TPGLs associated with multifocal head and neck PGLs.
- Germline testing for mutations associated with pheochromocytoma/PGLs syndrome is indicated in patients diagnosed with TPGL.
- This case illustrates the importance of an experienced multidisciplinary team approach to the evaluation and management in complex and rare TPGL cases.

## Data Availability

Data sharing is not applicable to this article as no datasets were generated or analyzed during the current study.

## Abbreviations

EBRT: external beam radiation therapy
PET/CT: positron emission tomography/computed tomography
PGL: paraganglioma
SDHD: succinate dehydrogenase complex subunit D
TN: thyroid nodule
TPGL: thyroid paraganglioma
